# Nitrogen-Fixing Gamma Proteobacteria *Azotobacter vinelandii*—A Blueprint for Nitrogen-Fixing Plants?

**DOI:** 10.3390/microorganisms12102087

**Published:** 2024-10-18

**Authors:** Sayre Barron, Florence Mus, John W. Peters

**Affiliations:** Department of Chemistry and Biochemistry, University of Oklahoma, Norman, OK 73019, USA

**Keywords:** nitrogen fixation, NifLA, Fix, Rnf, plant engineering

## Abstract

The availability of fixed nitrogen limits overall agricultural crop production worldwide. The so-called modern “green revolution” catalyzed by the widespread application of nitrogenous fertilizer has propelled global population growth. It has led to imbalances in global biogeochemical nitrogen cycling, resulting in a “nitrogen problem” that is growing at a similar trajectory to the “carbon problem”. As a result of the increasing imbalances in nitrogen cycling and additional environmental problems such as soil acidification, there is renewed and increasing interest in increasing the contributions of biological nitrogen fixation to reduce the inputs of nitrogenous fertilizers in agriculture. Interestingly, biological nitrogen fixation, or life’s ability to convert atmospheric dinitrogen to ammonia, is restricted to microbial life and not associated with any known eukaryotes. It is not clear why plants never evolved the ability to fix nitrogen and rather form associations with nitrogen-fixing microorganisms. Perhaps it is because of the large energy demand of the process, the oxygen sensitivity of the enzymatic apparatus, or simply failure to encounter the appropriate selective pressure. Whatever the reason, it is clear that this ability of crop plants, especially cereals, would transform modern agriculture once again. Successfully engineering plants will require creating an oxygen-free niche that can supply ample energy in a tightly regulated manner to minimize energy waste and ensure the ammonia produced is assimilated. Nitrogen-fixing aerobic bacteria can perhaps provide a blueprint for engineering nitrogen-fixing plants. This short review discusses the key features of robust nitrogen fixation in the model nitrogen-fixing aerobe, gamma proteobacteria *Azotobacter vinelandii*, in the context of the basic requirements for engineering nitrogen-fixing plants.

## 1. Introduction

Nitrogen availability is the main limiting factor of agricultural productivity, so synthetic nitrogen fertilizers have been introduced to increase yields. These additions ensure food security but are costly and negatively affect the environment by damaging sensitive ecosystems, reducing air quality, and contributing to the nitrogen problem [1,2]. The addition of fertilizer increases the amount of reactive nitrogen in the soil. However, when rates of nitrogen addition and denitrification are unequal, the excess is transported to bodies of water, resulting in eutrophication [3,4]. This scenario is comparable to excess anthropogenic carbon dioxide production beyond the amount that can be used by plants or buried. The nitrogen problem has led to an increased interest in biological nitrogen fixation (BNF) in diazotrophs. Diazotrophs are responsible for an estimated 60% of all fixed nitrogen and are found free-living in soil, the rhizosphere of plants, or in the N-fixing nodules of legumes [5,6,7]. In the absence of other nitrogen sources, these organisms can convert nitrogen gas to ammonia enzymatically and release ammonia for use by crop plants. Increasing the productivity of BNF would help reduce the need for synthetic fertilizers and decrease the negative impacts of their use [8].

Nitrogen fixation is performed by many types of bacteria and archaea using nitrogenase [9,10]. Nitrogenase contains oxygen-sensitive iron-sulfur clusters and requires high amounts of ATP and low-potential electrons to reduce N_2_. Across diazotrophs, many mechanisms have been developed to ensure an oxygen-free environment for nitrogenase [11,12,13,14]. Some organisms create physical barriers to create compartments free of oxygen [15]. Obligate anaerobes and facultative anaerobes fix nitrogen in the absence of oxygen [16,17]. Photosynthetic diazotrophs fix nitrogen when photosynthesis is not being performed [12,16,18,19]. Aerobes have high respiration rates of consuming oxygen, which allows them to protect nitrogenase [17,20]. Though oxygen presence is detrimental to nitrogenase, oxygen is beneficial in aerobes, allowing greater ATP production [20]. The higher amounts of ATP produced in aerobes allow higher levels of N_2_ reduction than by anaerobic organisms.

Eukaryotic plants and algae have not evolved to have the ability to fix nitrogen; perhaps they have not had the right selective pressures to incorporate nitrogen fixation machinery [21]. There is significant interest in using synthetic biology and genetic engineering to transfer the ability to fix nitrogen from bacteria to plants or algae [22,23]. A potential solution is to engineer the mitochondria to express nitrogenase and cofactor biosynthesis proteins and so far has been carried out in yeast [24,25,26,27,28,29,30]. Like aerobic diazotrophs, the mitochondria produce high levels of ATP and consume oxygen. However, in addition to its complex environmental requirements, nitrogenase needs many components for assembly [31,32]. Regulatory systems would also be necessary to ensure that energy requirements and ammonia produced would be balanced with plant needs. *Azotobacter vinelandii* is an aerobic diazotroph that meets the needs of nitrogenase and can serve as a model for how to engineer the mitochondria to fix nitrogen effectively in the presence of oxygen. This short review outlines the features of the metabolism of *A. vinelandii* that could be employed as a blueprint for engineering nitrogen fixation into the mitochondria toward the goal of more sustainable agriculture. While the direct incorporation of these features into the mitochondria may be difficult, we hope to highlight the utility of these systems in *A. vinelandii* and that the role they play is vital to balancing nitrogen fixation with normal metabolic processes.

In order to engineer nitrogen fixation into the mitochondria, a number of essential requirements must be met [33]. One of the foremost requirements, which at first approximation seems unachievable in oxygenic phototrophs, is a niche free of oxygen. The second is meeting the high energy demand of nitrogen fixation since nitrogenase requires 16 equivalents of MgATP for each dinitrogen reduced. A third requirement is a source of low-potential electrons since eight electrons are required to reduce dinitrogen, and electrons with a reducing potential of NADH are inadequate for nitrogenase catalysis [34]. The fourth is high fidelity regulation that responds to energy status, carbon/nitrogen ratio, and oxygen presence [35]. *A. vinelandii* is a well-studied model diazotroph that has an inherent high rate of respiration, which is (1) an inspiration for the idea that one could successfully express active nitrogenase in the mitochondria and (2) the metabolic blueprint for implementing such a herculean feat.

## 2. *A. vinelandii* as a Blueprint

*A. vinelandii* possesses a fascinating mechanism that protects the nitrogen fixation apparatus from oxygen while enabling robust nitrogen fixation. The “respiratory protection mechanism”, as it is termed, takes advantage of alternative paths of electrons through the respiratory chain to modulate the rates of oxygen reduced with the amount of proton force available for ATP production [36]. Typically, electrons enter the electron transfer chain through respiratory complex I, where the oxidation of NADH is coupled to the reduction of quinone to quinol. Electrons are transferred in a similar manner through the remainder of the respiratory chain, where complex III oxidizes quinol and reduces cytochrome c, which is subsequently oxidized by complex IV coupled to the reduction of oxygen to water. The free energy release through the transfer of electrons down an electrochemical potential gradient in all three complexes (I, III, and IV) is coupled to the translocation of protons from the mitochondrial matrix of the microbial cell cytoplasm to the inner membrane space or microbial periplasm. The translocation of protons generates a proton gradient, or proton motive force, to produce ATP.

**Figure 1:**
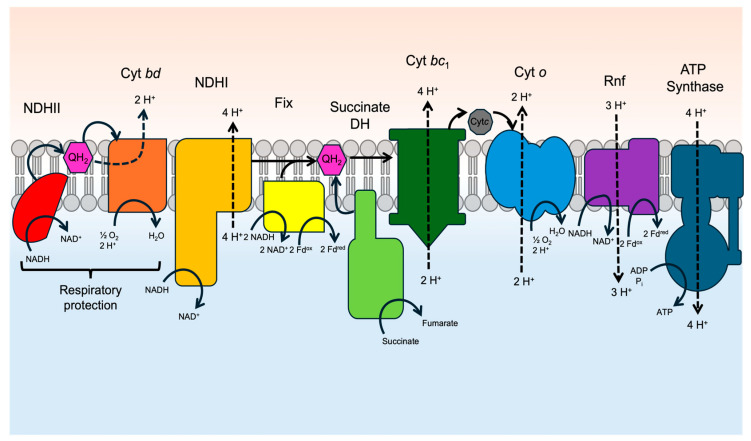
*A. vinelandii* respiratory protection, electron transport chain, and low-potential electron mechanisms. NDHII and Cyt *bd* are additional machinery used to increase *A. vinelandii*’s oxygen consumption. This occurs through the oxidation of NADH and subsequent reduction of quinone. The electrons are then transferred to Cyt *bd* where 2 protons are released across the membrane and 2 protons are used to form water. The typical electron transport chain consists of NDHI, Cyt *bc1*, Cyt *c*, and Cyt *o*. NDHI oxidizes NADH and transfers electrons to quinone while pumping out 4 protons. Quinone then passes electrons to Cyt *bc1,* then Cyt *c,* then Cyt *o,* where oxygen is reduced. Fix and Rnf reduce ferredoxin or flavodoxin using electrons from NADH. Solid lines indicate the movement of electrons, and dashed lines show the proton movement.

**Figure 2:**
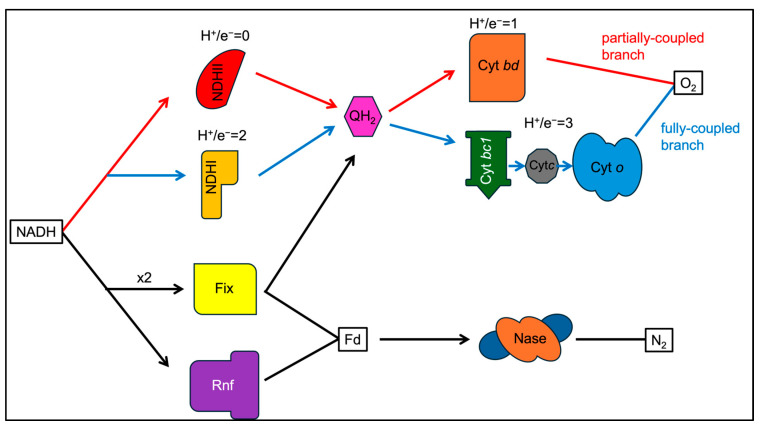
Electron transport paths. Electrons from NADH can go through one of 4 paths. The first branch (shown in red) is the partially coupled branch involving NDHII and Cyt *bd,* where electron transport is only coupled to proton translocation in Cyt *bd*. The fully coupled branch (shown in blue) utilizes the typical electron transport chain machinery to pump protons out. The other two paths involve Fix and Rnf (shown in black), which generate low-potential electrons using electron bifurcation or proton motive force, respectively.

The respiratory chain of *A. vinelandii* includes the prototypical complexes but is supplemented with alternatives to complex I (NDHII) and complex IV (Cyt *bd*) (Figure 1) [37,38,39,40]. These alternative complexes are capable of oxidizing NADH and reducing O_2_ at higher rates than the standard complexes, but only the activity of Cyt *bd* translocates protons and contributes to the proton motive force (Figure 2). Modulating the expression and activity of the entire suite of respiratory complexes allows for high fidelity control of the rate of O_2_ reduction to protect nitrogenase from oxygen damage [37]. The respiratory protection mechanism is really the inspiration for engineering nitrogen fixation into the mitochondria of plants, where the large respiratory capacity of the organelle should be able to provide a similar reducing environment for maintaining active nitrogenase while at the same time regulating the production of ATP in concert.

**Figure 3:**
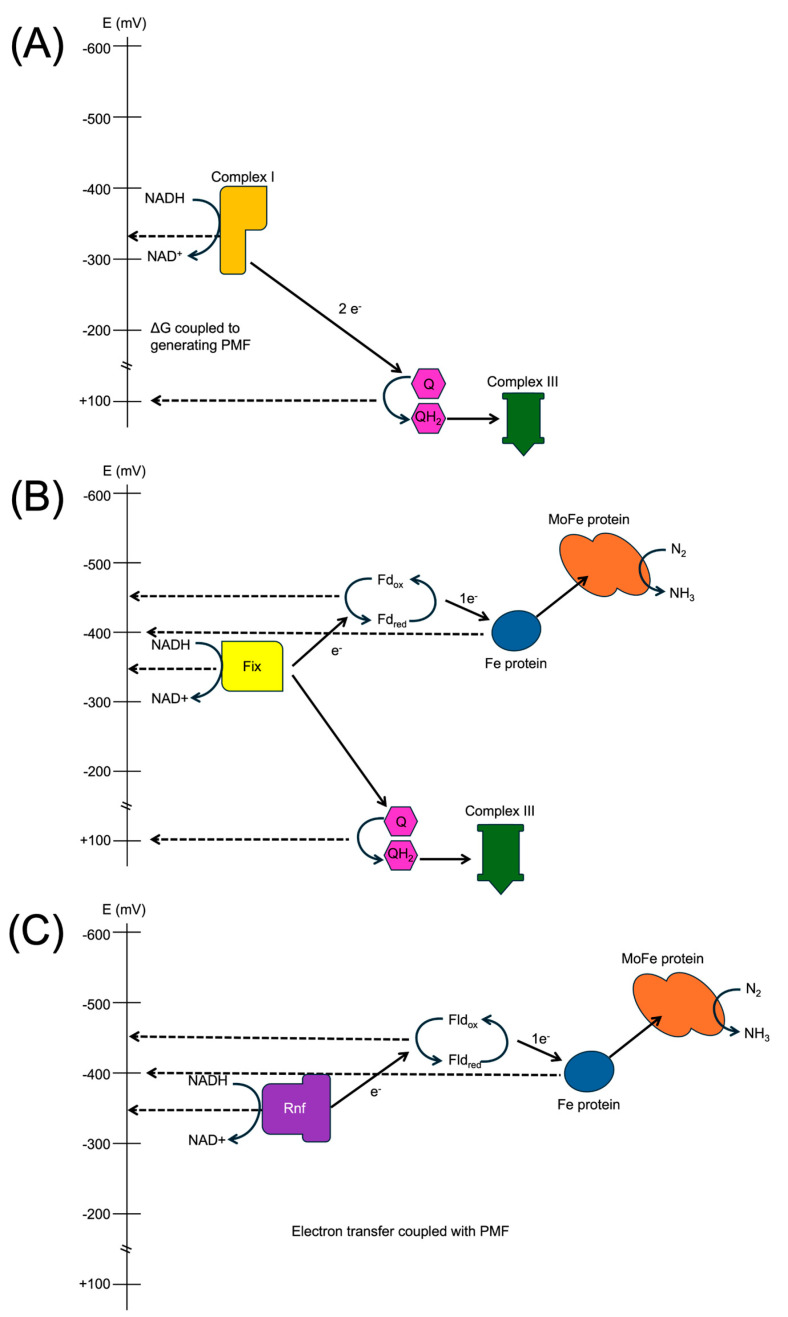
NADH utilization comparison. Solid arrows indicate electron flow. (**A**) Complex I oxidizes NADH and transfers electrons to reduce quinone, coupling with proton transport. (**B**) Fix utilizes electron bifurcation to transfer one electron to high-potential quinone and one electron to low-potential ferredoxin. (**C**) Rnf couples electron transfer with proton motive force to reduce flavodoxin.

Low-potential electrons are required for biological nitrogen fixation, and the standard currency of electrons in the mitochondria and *A. vinelandii* is NADH. NADH has an insufficient reduction potential for nitrogenase catalysis even at very high ratios of NADH to NAD^+^ where the reduction potential approaches ~−400 mV. Reduction potentials approaching −500 mV are required and provided by low-potential ferredoxin and flavodoxin [41]. NADH is the primary metabolic currency of reducing equivalents in *A. vinelandii*; however, there are two elegant mechanisms intimately coupled to the respiratory chain and under the control of nitrogen-fixation-specific gene expression (Figure 3) [41,42,43]. The first mechanism is encoded by the *Fix* gene locus, which is thus termed because it has a function related to nitrogen *fix*ation but is not formally part of the *nif* gene loci [44]. The FixABCX complex is a membrane-associated, heterotetrameric complex with multiple redox cofactors. This complex catalyzes an electron bifurcation reaction where the oxidation of NADH can result in the reduction of the more negative potential ferredoxin or flavodoxin when the reaction is stoichiometrically coupled to the reduction of the more positive potential quinone [43,45,46]. In this manner, half of an electron pair is diverted from the respiratory chain oxidation of NADH to the reduction of ferredoxin or flavodoxin, thereby impacting the flux of electrons to oxygen reduction and the generation of proton motive force. The second mechanism also diverts electrons from NADH away from oxygen reduction and the generation of proton motive force and simultaneously results in the consumption of proton motive force. The enzyme responsible for this activity is encoded by the *Rnf* genes, which are so called because their activity was implicated to be ***r***elated to ***n***itrogen ***f***ixation function [47,48]. The enzyme complex catalyzes the oxidation of NADH and the reduction of ferredoxin coupled to proton translocation from the periplasm to the cytoplasm. This exergonic translocation of protons provides the necessary free energy to drive the endergonic reduction of ferredoxin by NADH [49]. Deletion mutant analysis has shown that *A. vinelandii* can tolerate the deletion of either the FixABCX or the Rnf complex with limited effects under growth conditions [50]. However, the deletion of both mechanisms results in a *nif*-phenotype of *A. vinelandii* strains incapable of diazotrophic growth, indicating the requirement of these systems for low-potential electron generation for nitrogen fixation [43].

Respiratory protection and the activities of FixABCX and Rnf in generating low-potential electrons are two key factors that make nitrogen fixation possible in obligate aerobes. However, these additions add to the energy burden of metabolism. When you layer on the requirement of 16 ATP to reduce one N_2_ to two NH_3_ and H_2_, it is clear that there needs to be a highly coordinated and high-fidelity regulation system. This system must ensure that the amount of fixed nitrogen needed is balanced with oxygen reduction, generation and utilization of proton motive force, production of low-potential reducing equivalents, and production and utilization/hydrolysis of ATP. Looking forward to using these features of *A. vinelandii* to engineer mitochondria of crops plants for biological nitrogen fixation, it is clear without the appropriate coordinate regulation, the gene transfers would simply result in very sick plants and low crop yields [21,51].

**Figure 4:**
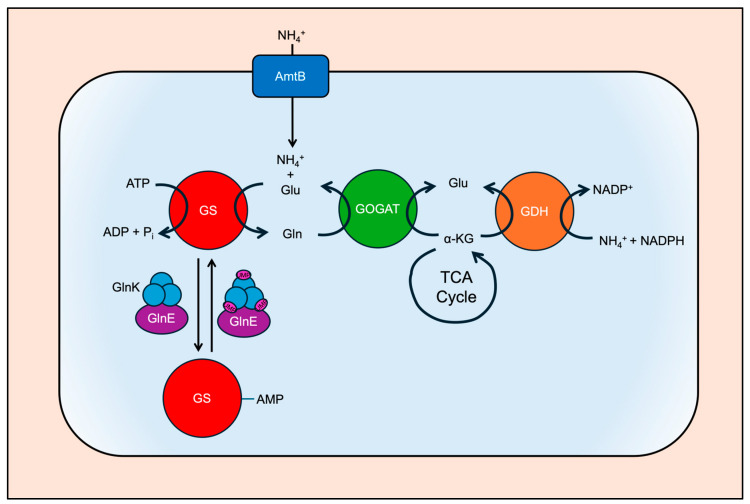
Nitrogen assimilation pathways in bacteria. Ammonium ions are transported into cells through Amt B, and it can then be utilized by either glutamine synthetase (GS) or glutamate dehydrogenase (GDH) to produce glutamine or glutamate, respectively. Glutamine can be utilized as a nitrogen donor to α-ketoglutarate (α-KG) to produce glutamate. Glutamine synthetase is reversibly modified by GlnE based on GlnK’s uridylyation status. In plants, ammonia is assimilated in the cytoplasm or chloroplast by GS. Ammonia can be uptaken by the roots and, in some species, produced in the mitochondria from photorespiration and proposed to passively move to the cytoplasm.

The expression of nitrogen fixation (*nif*) genes is controlled in response to several factors and is intimately linked to the regulation of nitrogen assimilation. Fixed nitrogen, usually ammonium ions or ammonia available in the cell’s environment, is then assimilated into amino acids. Many organisms, including bacteria and plants, use common machinery for the incorporation of fixed nitrogen, or nitrogen assimilation (Figure 4). Once imported by an Amt transporter, ammonium ions can then be assimilated by one of two different enzymatic paths [52]. The first path proceeds via the coupled reactions of glutamine synthase and glutamate synthase (also known as glutamine oxoglutarate aminotransferase), often termed GS-GOGAT. GS catalyzes the ATP-dependent incorporation of ammonium ions into glutamate to form glutamine. Then in sequence, GOGAT catalyzes the transfer of an amino group from resulting glutamine to α-ketoglutarate to form two glutamates [53,54,55]. The second enzymatic path is catalyzed by a single enzyme, glutamate dehydrogenase (GDH). GDH catalyzes the incorporation of ammonium ions onto α-ketoglutarate to form glutamate in a redox-dependent manner resulting in the oxidation of NADPH [56,57]. Though *A. vinelandii* contains a gene annotated as GDH, the GS-GOGAT has been shown to be the primary pathway for nitrogen assimilation [58]. Plants contain isoforms of GS, GOGAT, and GDH based on the cell location and their role in one of three assimilation pathways: primary assimilation, reassimilation of photorespiratory ammonia, and reassimilation of recycled nitrogen. In general, GS-GOGAT enzymes are used primarily due to GDH’s low affinity for ammonia [59,60,61].

The expression of nitrogen assimilation genes is regulated by the NtrBC two component regulatory system. NtrB is a sensor histidine kinase and responds to nitrogen status through the activity of the PII protein, GlnB. Under nitrogen-limiting conditions, Gln B interactions stimulate NtrB phosphatase activity resulting in autophosphorylation on the conserved histidine in an ATP-dependent reaction where one monomer phosphorylates the other [62,63]. This process activates and phosphorylates NtrC, a σ^54^-dependent response regulator [64,65,66]. Under nitrogen-sufficient conditions, NtrB inactivates NtrC through dephosphorylation.

NtrC is a σ^54^-dependent response regulator with three domains [67,68,69,70]. The N-terminal domain acts as the receiver domain containing a conserved aspartate phosphorylation site. The central domain contains the nucleotide binding site, and the C-terminal domain contains a helix-turn-helix motif for DNA binding. When the aspartate residue on the N-terminal domain is phosphorylated, NtrC is competent to activate transcription; this induces oligomerization at upstream activator sequences and ATPase activity [71,72]. The ATPase activity, in combination with the interaction of the central domain with the sigma factor-containing RNA polymerase, activates transcription [73,74,75]. The helix-turn-helix motif of the C-terminal domain mediates DNA binding of the upstream activator sequence where the RNA polymerase binds [76,77]. NtrC’s DNA binding properties allow it to act as a transcriptional repressor when not phosphorylated.

In some nitrogen-fixing organisms, NtrBC is directly involved in the regulation of nitrogen fixation (*nif*) gene expression, but *A. vinelandii* has another layer of regulation of nitrogen fixation specific regulation that has some features in common with the NtrBC regulatory mechanisms but also some interesting twists of the two-component regulatory paradigm. The NifLA two-component regulatory system is constitutively expressed, and it regulates nitrogen fixation gene expression in response to redox status (oxygen), energy status (ATP/ADP ratios), and nitrogen status through crosstalk with the nitrogen assimilation apparatus [11].

**Figure 5:**
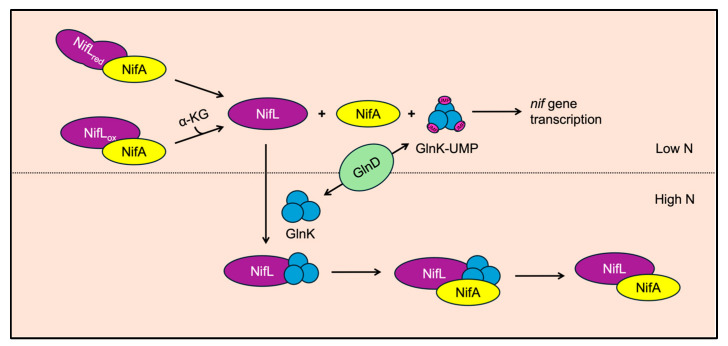
NifLA system mechanism. To activate *nif* gene expression, NifA cannot be interacting with NifL. This complex dissociates when NifL is reduced or when NifA is saturated by α-ketoglutarate (a-KG). When nitrogen levels in the cell are low, GlnK is uridylylated by GlnD and unable to bind NifL. When nitrogen levels are high, GlnK is de-uridylyated and binds NifL, stimulating the formation of the NifLA-GlnK complex.

In Proteobacteria, including *A. vinelandii*, nitrogen fixation (*nif*) gene expression is controlled by NifLA, which is an interesting variation of the two-component regulatory system paradigm [35,78,79]. NifA is a σ^54^-dependent transcriptional activator that stimulates the expression of nif genes [80,81,82,83,84]. NifL is homologous to sensor histidine kinases (SHKs) like NtrB. However, NifL does not hydrolyze ATP and does not function as a kinase/phosphatase like NtrB. It is also not subject to phosphorylation in its role in modulating NifA activity [84]. NifL undergoes conformational changes in response to cellular signals of oxygen, energy, and fixed nitrogen status and modulates NifA activity by binding or releasing NifA (Figure 5) [85].

NifL senses these cellular cues and propagates the signal via its four discrete domains, similar to class I SHKs [84,86,87,88]. The amino-terminal (N-terminal) portion of NifL contains tandem PAS domains, PAS1 and PAS2. The PAS1 domain includes a solvent-accessible FAD cofactor that is readily oxidized by intracellular oxygen and is the only part of NifL with a known structure [89,90,91]. Oxidation of the PAS1 FAD causes reorganization of hydrogen bonds within the FAD-binding pocket. These changes lead to a reorientation of the non-FAD-containing PAS2 domain to stimulate NifL binding to NifA, inhibiting nitrogenase expression in oxidizing conditions [92,93,94,95]. The carboxy-terminal (C-terminal) kinase-like DH and GHKL domains perceive energy and fixed nitrogen signals [96]. The NifL DH domain is homologous to the dimerization and histidine phosphotransfer (DHp) domain that contains the conserved His in SHKs, and the GHKL domain closely resembles the catalytic domain of canonical SHKs [96,97]. However, NifL does not function either as a kinase or a phosphatase. Rather than hydrolyzing ATP to catalyze phosphorylation, the GHKL domain binds adenosine nucleotides ADP and ATP, sensing the cellular energy status via the ADP/ATP ratio and assuming a NifA-binding conformation when bound to ADP. NifL exhibits a 10-fold higher affinity for ADP than ATP, which ensures that nitrogenase is only expressed in energy-rich conditions that can support the energetic demands of nitrogenase [88]. Similar to the sensor kinase domain of NtrB, NifL also perceives nitrogen status through interactions with the PII protein GlnK [98,99,100].

As a member of the PII protein family, GlnK is post-translationally modified by GlnD based on the α-ketoglutarate/glutamine ratio in the cell [100]. The uridylylation state of GlnK modulates interactions with NifL; GlnK is reversibly uridylylated by GlnD. In low-nitrogen conditions, GlnK is uridylylated and it is unable to interact with NifL. Once deuridylylated in high-nitrogen conditions, GlnK can interact with NifL and stimulate the formation of the NifLA complex [98]. Though the interactions between NifL and GlnK and the role of alpha-ketoglutarate and nucleotides in these interactions are not clearly understood, it is clear that GlnK plays an important role in communicating information about the cell’s fixed nitrogen status.

NifLA’s regulation is robust and can respond to elements needed to identify optimal conditions for nitrogen fixation in bacteria, which suggests that it should be effective at responding to these elements in plants to ensure the process is balanced with the plant metabolism. It is unclear whether there will be compatibility with the plant’s nitrogen assimilation machinery and NifLA’s regulation; however, NifLA’s mechanism for sensing carbon/nitrogen status will compensate for incompatibility. The role of GlnE in nitrogen assimilation is to promote the inactivation of glutamine synthetase (GS) based on the uridylylation status of GlnK. In the absence of GlnE, GS is always active, and glutamine is continuously produced in the presence of ammonium ions. Previous work in microbial systems that do not fix nitrogen has shown that the deletion of GlnE results in growth limitations in the presence of excess ammonium ions [101,102]. This has been interpreted to result from the absence of GS regulation and the consumption of α-ketoglutarate, resulting in a stall in carbon metabolism. In our previous work, we have shown that when GlnE is deleted in *A. vinelandii,* an analogous phenotype is observed when grown under nitrogen-replete conditions, but growth in diazotrophic conditions overcomes this deleterious growth phenotype [103]. We attributed this growth phenotype to previous observations that NifA can bind α-ketoglutarate and that binding regulates NifA’s ability to promote the transcription of *nif* genes. These results indicate that the NifLA system has its own high-fidelity sensor of carbon/nitrogen status and is not dependent on the crosstalk with the nitrogen assimilation regulatory machinery.

## 3. Conclusions

**Figure 6:**
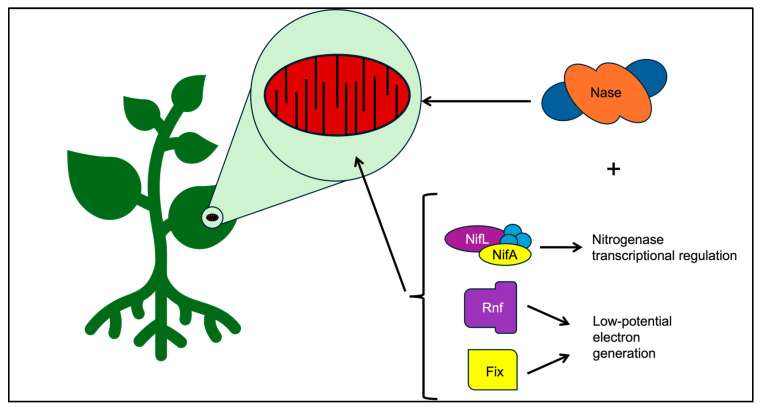
Summary of proposed components based on *A. vinelandii* as a blueprint. *A. vinelandii* contains NifLA, Rnf, and Fix systems that ensure the needs of nitrogenase are met. NifLA regulates transcription of nitrogenase based on cellular energy levels, oxygen presence, and nitrogen levels. Rnf and Fix produce low-potential electrons for use by nitrogenase.

The quest for engineering nitrogen fixation plants has several perceived barriers, and probably some that still need to be realized. The perceived barriers include providing an appropriate niche in a plant where the nitrogen fixation enzymatic machinery can be protected from oxygen and where there are ample energy reserves to accomplish energy-intensive nitrogen reduction. The mitochondrion is a niche that can satisfy these requirements, and nitrogenase and cofactor synthesis proteins have been successfully expressed and targeted for localization in the mitochondria of yeast [29,30]. Still, several modifications are needed to convert it to a nitrogen-fixing organelle in a manner that does not simply result in severe growth limitations. *A. vinelandii* utilizes a high respiration rate, membrane-associated complexes for low-potential electron generation, and robust transcriptional regulation to fix nitrogen efficiently in the air, making it an ideal blueprint for several features (Figure 6).
